# Material Hardship in Families With Low Income: Positive Effects of Coparenting on Fathers’ and Mothers’ Parenting and Children’s Prosocial Behaviors

**DOI:** 10.3389/fpsyg.2021.729654

**Published:** 2021-12-09

**Authors:** Joyce Y. Lee, Brenda L. Volling, Shawna J. Lee

**Affiliations:** ^1^College of Social Work, The Ohio State University, Columbus, OH, United States; ^2^Department of Psychology, University of Michigan, Ann Arbor, MI, United States; ^3^School of Social Work, University of Michigan, Ann Arbor, MI, United States

**Keywords:** Building Strong Families, Family Stress Model, risk and resilience framework, material hardship, coparenting alliance, responsive mothering and fathering, children’s prosocial behaviors

## Abstract

Families with low income experience high levels of economic insecurity, but less is known about how mothers and fathers in such families successfully navigate coparenting and parenting in the context of material hardship. The current study utilized a risk and resilience framework to investigate the underlying family processes linking material hardship and children’s prosocial behaviors in a sample of socioeconomically disadvantaged mother-father families with preschoolers from the Building Strong Families project (*N* = 452). Coparenting alliance and mothers’ and fathers’ responsive parenting were examined as mediators. Results of structural equation modeling showed that coparenting alliance was associated with higher levels of both mothers’ and fathers’ responsive parenting. Subsequently, both parents’ responsive parenting were associated with higher levels of children’s prosocial behaviors. Material hardship was not associated with coparenting alliance and either parent’s responsive parenting. Tests of indirect effects confirmed that the effects of coparenting alliance on children’s prosocial behaviors were mediated through both mothers’ and fathers’ responsive parenting. Overall, these results suggest that when mothers and fathers have a strong coparenting alliance, they are likely to withstand the negative effects of material hardship and thus engage in positive parenting behaviors that benefit their children’s prosocial development. Family strengthening interventions, including responsible fatherhood programs, would do well to integrate a strong focus on enhancing a positive coparenting alliance between mothers and fathers.

## Introduction

Material hardship—defined as challenges with paying for food, housing, utilities, or medical care—is prevalent among American families with low income, with 70% of such families reporting some level of material hardship (Ouellette et al., 2004; Karpman et al., 2018). Although empirical evidence on the effects of material hardships on family functioning is more limited than those of income poverty material hardship has been linked with negative family and child outcomes, including lower levels of interparental relationship quality (Lucas et al., 2020), less sensitive parenting (Newland et al., 2013), and children’s lower cognitive skills and socioemotional competence (Gershoff et al., 2007). That said, less is known about the family process underlying some of these links in two-parent families with low income and whether resilience present in such families buffers the negative effects of material hardship on relevant family processes and ultimately children’s development. Thus, the current study aimed to utilize a risk and resilience framework to understand underlying family processes (e.g., coparenting and parenting) linking material hardship and young children’s prosocial behaviors using data from the Building Strong Families (BSF) project, a large and racially diverse sample of socioeconomically disadvantaged mother-father families with low income.

### Theoretical Framework: The Family Stress Model

The Family Stress Model (FSM: Conger et al., 1992) was first devised to understand better the impact of negative economic events on families in the Midwestern United States during the Great Farm Crisis in the 1980s. The earliest FSM studies used samples of White families in rural farming communities in Iowa (Conger et al., 1990, 1992, 1993, 1994) and showed that negative economic events were associated with poor outcomes for children mainly through their effects on parents’ mental health, relationship quality, and parenting behaviors. Specifically, the FSM posits that economic pressures arising from negative economic events such as low family income, income loss, unstable work, or debts can lead to higher levels of depressive moods for both mothers and fathers, which then lead to relationship strain in the form of interparental conflict. Subsequently, poor interparental relationship quality is linked to lower involved or nurturant parenting behaviors that ultimately result in children’s maladjustment (Conger et al., 1992).

Expanding on this work, researchers have also tested the FSM with racially diverse samples and have found support for the model (Conger et al., 2002; Parke et al., 2004; Masarik and Conger, 2017; Gard et al., 2020; Curran et al., 2021; Lee et al., 2021). For example, Lee et al. (2021) recently applied the FSM to a sample of BSF families and found that fathers’ depressive symptoms was a mediating path between material hardship, but not income poverty, and destructive interparental conflict. Curran et al. (2021) also applied the FSM to a BSF sample and showed in cross-lagged panel models that fathers’ depressive symptoms at the 15-month follow-up predicted higher levels of destructive interparental conflict at the 36-month follow-up, but not vice versa. Both studies underscore the centrality of paternal mental health as a significant factor affecting family processes, namely interparental relationship quality.

Neither BSF study, however, included parenting nor child outcomes, and more importantly, both focused on testing the FSM looking at family conflict and poor mental health and did not use a risk and resilience framework and consider the buffering effects of positive family dynamics. The current study was designed to test how a supportive coparenting alliance between mothers and fathers predicted responsive parenting and in turn, children’s prosocial behavior in an effort to look at protective factors within families experiencing material hardship.

### Material Hardship to Coparenting Alliance and Mothers’ and Fathers’ Responsive Parenting

Prior studies have examined the links between material hardship, coparenting alliance, and responsive parenting behaviors (Gershoff et al., 2007; LeBaron et al., 2020; Curran et al., 2021). Coparenting alliance is often characterized by both parents’ investments in their children, a respect for each other’s judgment about child rearing, and a desire to communicate child-related information (Weissman and Cohen, 1985; Feinberg, 2003). Recently, LeBaron et al. (2020) used a sample of two-parent families from the BSF project and showed that material hardship at the 15-month follow-up was linked with lower levels of fathers’ perceived coparenting alliance (i.e., communication, support, and teamwork), but not mothers’ perceived coparenting alliance, at the 36-month follow-up. The researchers noted the possibility that when fathers with low income are faced with financial strain that makes it difficult to help meet their families’ material needs, they may end up prioritizing financially providing for their families over building a coparenting alliance with mothers (LeBaron et al., 2020). That is, stress with meeting their families’ material needs may undermine socioeconomically disadvantaged fathers’ abilities to successfully engage in positive coparenting behaviors with their partners. Alternatively, mothers may be more likely to engage in gatekeeping behaviors when fathers do not meet breadwinner norms (e.g., unemployed) (Waller, 2012) and the financial stress associated with material hardship and meeting the needs of the family may take its toll on the coparenting relationship. Unlike LeBaron et al. (2020) though, Curran et al. (2021) in their cross-lagged modeling of material hardship and coparenting alliance using BSF data found that material hardship at the 15-month follow-up was not associated with either mothers’ or fathers’ perceived coparenting alliance at the 36-month follow-up.

Findings on material hardship and responsive parenting also seem to be mixed, and available studies seem to primarily focus on mothers. In one study examining links between material hardship and mothers’ positive parenting, Shelleby (2018) used data from the Fragile Families Child Wellbeing Study (FFCWS) and found that material hardship when children were a year old was not linked with mothers’ positive parenting (e.g., praise child, warmth) when children were 5 years old. They did not include information on fathers, even though work cited earlier suggested that men’s mental health was a contributing factor to family conflict. Gershoff et al. (2007) also focused predominantly on mothers by using data from the Early Childhood Longitudinal Study-Kindergarten cohort (ECLS-K) and found that material hardship was linked with higher levels of maternal positive parenting (e.g., warmth, cognitive stimulation) when children were 6 years old—a finding that was unexpected. The researchers noted that mothers may be investing in positive parenting behaviors, when they are unable to provide economic resources to improve their children’s lives. Few studies focus specifically on material hardship and fathers’ positive parenting, and instead use indicators of fathers’ economic conditions (e.g., employment status, living in poverty) to examine relations between fathers’ parenting and children’s outcomes (Johnson, 2001; Waller, 2012; Baker et al., 2018). For example, using a sample of fathers from the FFCWS, Waller (2012) showed that fathers being employed when their children were 3 years old was associated with mothers’ reports of fathers spending more time with their children but fathers engaging in a lower number of daily activities (e.g., playing outside, reading stories, and singing songs).

When studies do include both mothers and fathers from socioeconomically disadvantaged backgrounds, which again are limited in number, there is evidence that lack of economic resources can negatively affect the quality of parent-child relationships. For instance, Baker et al. (2018) examined fathers and mothers from the Early Childhood Longitudinal Study-Birth Cohort (ECLS-B) and showed that poverty levels were related to lower levels of paternal warmth and cognitive stimulation during fathers’ interactions with their 24-month-old children in the home. Family poverty was associated only with lower levels of cognitive stimulation during mother-child interactions. Overall, given the mixed results of prior research and limited number of studies including both mothers and fathers, additional research is needed to understand better the links between material hardship, the coparenting alliance, and mothers’ and fathers’ responsive parenting among families with low income.

### Coparenting Alliance and Children’s Prosocial Behaviors via Mothers’ and Fathers’ Responsive Parenting

Research has examined relations specifically between the coparenting alliance and positive parenting behaviors for both mothers and fathers from socioeconomically disadvantaged backgrounds (Jones et al., 2005; Shook et al., 2010; Barnett et al., 2011; Fagan and Palkovitz, 2011; Lee et al., 2020). For example, Barnett et al. (2011) used a community sample of mothers whose children were enrolled in Head Start and found that mothers’ reports of a supportive coparenting alliance predicted maternal warmth with their 4-year-old children. In a study with mothers and fathers from the FFCWS, Fagan and Palkovitz (2019) showed that mothers’ reports of a supportive coparenting alliance when children were a year old predicted higher levels of fathers’ engagement (e.g., read stories, sing songs, play) when the children were 3 years old. Recently, Lee et al. (2020) used BSF data and found that a supportive coparenting alliance between mothers and fathers at the 15-month follow-up predicted higher levels of fathers’ engagement in caregiving such as clothing and feeding at the 36-month follow-up, but only for residential fathers.

### Positive Parenting and Children’s Prosocial Development

Mothers’ and fathers’ positive parenting behaviors—such as being sensitive to the needs of the child and displaying warmth—are linked with children’s development of prosocial behaviors starting in early childhood (Grusec et al., 2002; Davidov and Grusec, 2006; Eisenberg et al., 2006; Hastings et al., 2007; Biringen and Easterbrooks, 2012; Brownell et al., 2013). Children’s prosocial behaviors include showing concern for others and a willingness to help or share with others. Although much of this research has been conducted with middle-class families, several studies have tested similar relations among families with low income. For example, using a community sample of families with low income, Barnett et al. (2012) found that maternal sensitivity was positively associated with prosocial behaviors when children were 24–36 months old. Studies examining fathers’ contributions—especially those from socioeconomically disadvantaged backgrounds—to young children’s prosocial behaviors are limited. Of the few available studies, Newton et al. (2014) using data from the National Institute of Child Health and Human Development Study of Early Child Care (NICHD-SECC) reported that both paternal and maternal sensitivity during structured observational tasks when children were 54 months old positively predicted children’s prosocial behaviors when they were 9 years old. Unfortunately, Newton et al. (2014) conducted separate analyses for mothers and fathers, rather than taking the interdependence between mothers and fathers into consideration and modeling the joint contribution of mothers and fathers to children’s prosocial development.

Because research suggests that girls generally engage in more prosocial behaviors than boys (Rose and Rudolph, 2006; Baillargeon et al., 2011; Kornbluh and Neal, 2014), we also considered children’s gender as a moderator of the paths between responsive parenting and children’s prosocial behaviors. Rose and Rudolph (2006) found gender differences in children’s prosocial behaviors in their review of the literature. Specifically, girls were consistently more prosocial than boys, as reported by both peers and teachers, across the kindergarten, elementary, and middle school years. Research on younger children appears mixed, with Baillargeon et al. (2011) finding that preschool girls were more likely than boys to show prosocial behaviors (e.g., will try to help someone who has been hurt, comforts a child who is crying or upset) between 29 and 41 months, but Yeh et al. (2018) finding no significant differences between girls’ and boys’ prosocial behaviors (e.g., offering to help, being kind toward peers, cooperative with peers).

### The Current Study

**FIGURE 1:**
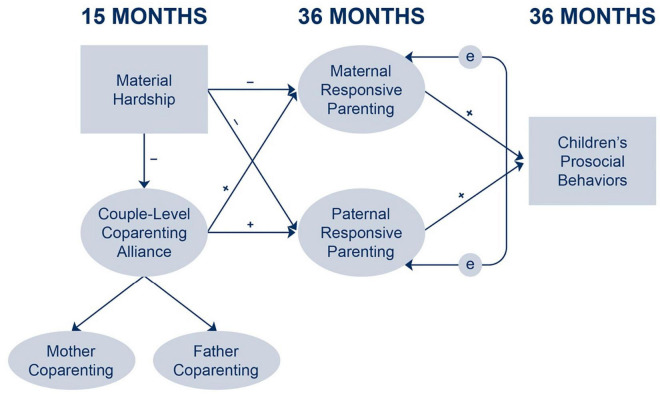
Conceptual model for the current study.

The current study aimed to utilize a risk and resilience approach to investigate the underlying family processes linking material hardship and children’s prosocial behaviors in a sample of socioeconomically disadvantaged mother-father families with preschoolers. Positive coparenting in the form of supportive alliance between mothers and fathers and responsive parenting were examined as mediators. There were three hypotheses based on the FSM and prior research (see Figure 1; Conger et al., 1994; Neppl et al., 2016; Gard et al., 2020; Lee et al., 2020). First, it was hypothesized that material hardship would be associated with a less supportive coparenting alliance at 15 months and less responsive parenting for both mothers and fathers at 36 months (H1). Second, a positive coparenting alliance would predict higher levels of mothers’ and fathers’ responsive parenting (H2). Finally, mothers’ and fathers’ responsive parenting would be associated with higher levels of children’s prosocial behaviors at 36 months and act as mediating pathways between coparenting alliance and children’s prosocial behaviors (H3) (Barnett et al., 2012; Newton et al., 2014). This study makes an important contribution to the literature by examining how positive family functioning, such as supportive coparenting alliance, may serve as a source of resilience to ultimately buffer the negative effects of material hardship on children’s socioemotional development, especially amongst children whose families experience poverty.

## Materials and Methods

### The Building Strong Families Project

Data came from the BSF project, a large-scale evaluation of a healthy marriage and relationship education program conducted between 2002 and 2013 across the United States, among romantically involved unmarried heterosexual couples, who were expecting or recently had a baby together (Wood et al., 2010). The project was funded by the U.S. Department of Health and Human Services and implemented by Mathematica Policy Research, with the goal to strengthen couples’ relationships and thus create healthy home environments for their children (Wood et al., 2014).

### Procedures

BSF recruited 5,102 couples from hospitals, prenatal clinics, and special nutritional programs for Women, Infants, and Children (WIC). Couples were eligible to enroll if (a) both the mother and father agreed to participate in the intervention; (b) the couple was romantically involved; (c) the couple was either expecting a baby together or had a baby younger than 3 months old; (d) the couple was unmarried at the time the baby was conceived; and (e) both parents were 18 years or older (Wood et al., 2010). Mathematica Policy Research obtained participants’ written consent and randomly assigned couples into either an intervention group (*n* = 2,553) or a control group (*n* = 2,549). The BSF intervention focused on providing 30–42 hours of relationship skills education to enrolled couples in the form of group sessions. The control group couples could seek relationship skills education from other sources but were not provided with the BSF intervention services. Data collection included three time points: (1) Baseline when couples enrolled in the project; (2) 15 months after enrollment via telephone surveys; and (3) 36 months after enrollment via telephone surveys. At the 36 month-follow up period, direct observations of mother-child and father-child interactions were also conducted in addition to telephone surveys. Children’s socioemotional developmental outcomes were only available at the 36-month follow-up period (see Moore et al., 2013 and Wood et al., 2014 for full details). The Health Sciences and Behavioral Sciences institutional review board at the University of Michigan approved the current study as secondary analysis of the BSF data (HUM00145063).

### Participants

The analytic sample consisted of BSF families in which both mothers and fathers had completed parent-child observations from the 36 month follow-up period, which was the time at which responsive parenting was assessed in the current study. The majority of such families (80–99% depending on which parent’s data were used) were residential in that both mothers and fathers reported living with each other and the focal BSF child. We further narrowed down our sample to families in which parents and the focal BSF child were consistently residential with each other across 15 and 36 months, the two times of measurement included in the current study. Aligned with prior research with socioeconomically disadvantaged families (Waller and Emory, 2014; Fagan et al., 2016; Lee et al., 2020), parental residential status was defined as living with one’s partner and child *all* or *most* of the time. Mothers and fathers reporting that they lived with each other or the focal BSF child some or none of the time were excluded. To create the analytic sample, of the 5,102 BSF families, 18 families with a deceased BSF partner were first excluded from the total sample. Second, 597 families from Baltimore were excluded because BSF only asked mothers and not fathers at this site to complete the parent-child observation sessions at the 36-month follow-up. Third, 3,314 families without observational data for both mothers and fathers were excluded. BSF collected observational data with majority residential families. Fourth, 517 families in which parents or the BSF child were not residential with each other at 15 months were excluded. Finally, another 204 families in which parents or the BSF child were not residential with each other at 36 months were excluded. The final analytic sample consisted of *N* = 452 families. Sample characteristics can be found in Table 1.

**TABLE 1:** Sample descriptive statistics.

Variable	*M* (*SD)* or %
Mothers’ age (range: 18–41 years)	23.75 (4.96)
Fathers’ age (range: 18–55 years)	26.29 (6.00)
Couples’ ethnicity and race:	
Black	35.5
White	29.78
Latinx	25.11
Other	9.56
Couples’ education:	
Neither parent has high school diploma	13.72
One parent has high school diploma	34.41
Both parents have high school diploma	52.88
Fathers’ employment status (Yes)	82.96
Fathers’ multiple-partner fertility (Yes)	27.43
Fathers’ involvement in caregiving^b^ (range: 1–6)^b^	4.19 (0.91)
Fathers’ depressive symptoms^a^ (range: 0–3)	0.26 (0.35)
Mothers’ depressive symptoms^a^ (range: 0–3)	0.36 (0.50)
Family material hardship^a^:	
Could not pay rent or mortgage	16.81
Utilities turned off because could not pay	7.52
Eviction from apartment or home	1.55
Lack of health insurance	91.81
Child sex (Boy)^a^	46.43
Assignment in the BSF program (Intervention)	52.65

*N = 452. Otherwise stated, all variables are from baseline when couples enrolled in the BSF program. BSF, Building Strong Families.*

*^a^Variable is from the 15-month follow-up period.*

*^b^Variable is from the 36-month follow-up period.*

### Measures

#### Material Hardship

Material hardship was a key independent variable and measured at the 15-month follow-up survey, using four items with dichotomous 0 = *No* or 1 = *Yes* responses: (1) *Ability to pay rent –* families’ hardship paying rent or mortgage in the past year (i.e., “You could not pay the full amount of the rent or mortgage?”); (2) *Consistency of utilities –* the hardship families experienced related to utilities in the past year (i.e., “You had services turned off by the water, gas, or electric company or the oil company would not deliver oil in the past 12 months because you could not afford to pay the bill?”); (3) *Residential stability* – the hardship families experienced related to housing in the past year (i.e., “You were evicted from your home or apartment because you could not pay the rent or mortgage?”); and (4) *Medical care –* the hardship families experienced related to medical insurance [e.g., “Are you currently covered by Medicaid, (STATE/LOCAL FILL), or any other government program that pays for medical care?”]. The medical care indicator was reverse coded with 1 indicating the presence of medical hardship with respect to insurance coverage. Although material hardship measures often include food insecurity as a relevant indicator of materials hardship, a food insecurity item was not available in the BSF dataset. Mothers’ reports were used primarily to create a variable indicating families’ material hardship although where data from mothers were missing, fathers’ reports were used. A total score was created by summing across all four items to create a composite of material hardship, ranging from 0 to 4.

#### Coparenting Alliance

Coparenting alliance between mothers and fathers was assessed at the 15-month follow-up survey and served as one of the mediating variables. Mothers’ and fathers’ reports of positive coparenting were measured using 10 items from the Parenting Alliance Index (PAI; Abidin and Brunner, 1995). The items represented a parent’s positive assessment—coparenting alliance and communication—of another parent as a coparent (e.g., “I believe my child’s other parent is a good parent,” “My child’s other parent and I communicate well about our child,” “I feel good about my child’s other parent’s judgment about what is right for our child,” “My child’s other parent makes my job of being a parent easier,” “My child’s other parent and I are a good team”). Fathers and mothers rated these items on a 5-point scale ranging from 1 = *strongly agree* to 5 = *strongly disagree*. The scale was reverse coded so that higher scores reflected higher levels of coparenting alliance. All 10 items served as individual indicators for fathers’ and mothers’ individual coparenting latent variables to be described later.

#### Parenting Behaviors

Mothers’ and fathers’ parenting behaviors observed at the 36-month direct assessment served as additional mediating variables. Parenting behaviors were observed and videotaped separately during the two-bags task, a 10-min semi-structured, free-play interaction task between a parent and child (Administration for Children and Families, 2002). The two-bags task is a modified version of the three-bags task (NICHD Early Child Care Research Network, 1999). Specifically, the task involved the interviewer placing a mat and two bags on the floor and asking the parent and child to spend time playing with objects in the two bags. The parent initially was instructed to open the first bag, which included a book inside, and then move on to the second bag, which included pretend play toys inside. The parent was further informed that he or she could divide the 10 minutes between the two bags as he or she wished. Eighteen trained coders rated six parenting behaviors from the parent-child interaction videos in a centralized location using the same rating system as the NICHD Study of Early Child Care Research Network (NICHD Early Child Care Research Network, 1999).

This rating system employs a 7-point scale ranging from 1 = *not at all characteristic* to 7 = *very characteristic* to code (a) *sensitivity*, which is the ability to perceive and accurately interpret the child’s behavior and respond appropriately; (b) *intrusiveness*, which pertains to interventions or overstimulation that impinges on the child’s independence; (c) *detachment*, which represents lack of involvement and disengagement with the child; (d) *positive regard*, which corresponds with demonstrating positive feelings toward the child; (e) *negative regard*, which corresponds to demonstrating negative feelings toward the child; and (f) *stimulation of cognitive development*, which involves scaffolding the child’s cognitive development during the task. All six parenting variables were used in the development of latent variables representing mothers’ and fathers’ responsive parenting.

#### Children’s Prosocial Behaviors

Children’s prosocial behaviors were assessed at the 36-month follow-up, using nine items from an adapted version of the Social Interaction Scale of the Preschool and Kindergarten Behavior Scales—Second Edition (PKBS-2; Merrell, 2002). The items represent young children’s prosocial behaviors (e.g., “Comforted other children who were upset”) in the last 3 months (Moore et al., 2013). Items from the PKBS-2 Social Interaction Scale have been adapted for use in large surveys, such as the Early Childhood Longitudinal Survey-Birth Cohort and Universal Preschool Child Outcome Study (Moore et al., 2013). Mothers rated the nine items on a 4-point scale, ranging from 1 = *often* to 4 = *never*. The scale was converted to range from 0 to 3, and the items were reverse averaged so that higher scores represented more prosocial behaviors (α = 0.76).

#### Sociodemographic Control Variables

A robust set of sociodemographic variables primarily from baseline were used as control variables in all the analytic models. These control variables were selected by examining related literature (Lee et al., 2020) and conducting correlations with the main study variables. Significant correlations were present between main study variables and the following 10 control variables: Couples’ race and ethnicity (White, Black, Latinx, other), couples’ education level (neither parent has a high school diploma, only one parent has a high school diploma, both parents have a high school diploma), couples’ relationship length, fathers’ employment status, mothers’ depressive symptoms, fathers’ depressive symptoms, fathers’ multiple partner fertility, fathers’ involvement in caregiving (composite of three items pertaining to feeding, diapering, and changing clothes), BSF random assignment status, and BSF program site location. All control variables were from baseline, except for mothers’ and fathers’ depressive symptoms, which were from the 15-month follow-up, and fathers’ involvement in caregiving, which was from the 36-month follow-up.

Specifically, mothers’ depressive symptoms (*r* = 0.14, *p* = 0.003) and fathers’ depressive symptoms (*r* = 0.19, *p <* 0.001) were positively correlated with family material hardship. Mothers’ depressive symptoms (*r* = −0.25, *p <* 0.001) were negatively correlated with mothers’ reports of coparenting alliance. Being Latinx (*r* = −0.12, *p* = 0.010), fathers’ depressive symptoms (*r* = −0.20, *p <* 0.001), and BSF program site location (*r* = −0.09, *p* = 0.049) were negatively correlated with fathers’ reports of coparenting alliance. Being randomly assigned to the BSF intervention group (*r* = 0.18, *p <* 0.001) was positively correlated with fathers’ reports of coparenting alliance. Fathers’ employment (*r* = 0.14, *p* = 0.003) and fathers’ multiple partner fertility (*r* = −0.10, *p* = 0.038) were positively and negatively correlated with mothers’ responsive parenting, respectively. Neither parent having a high school diploma (*r* = −0.12, *p* = 0.011) was negatively correlated with fathers’ responsive parenting, whereas both parents having a high school diploma (*r* = 0.10, *p* = 0.037) was positively correlated with fathers’ responsive parenting. Finally, being White (*r* = 0.10, *p* = 0.032), being Black (*r* = 0.17, *p <* 0.001), and both parents having a high school diploma (*r* = 0.14, *p* = 0.002) were positively correlated with children’s prosocial behaviors. Being Latinx (*r* = −0.32, *p <* 0.001), only one parent having a high school diploma (*r* = −0.11, *p* = 0.015), and couple relationship length (*r* = −0.13, *p* = 0.015) were negatively correlated with children’s prosocial behaviors.

### Model Development and Data Analysis Plan

Correlations between the main variables, including indicators of key factors, can be found in Table 2. Consistent with prior literature using BSF data, we used observed variables for material hardship (Curran et al., 2021; Lee et al., under review) and children’s prosocial behaviors (Love et al., 2009) and created latent variables for coparenting alliance (Lee et al., 2020) and mothers’ and fathers’ responsive parenting (Caughy et al., 2016).

**TABLE 2:** Descriptive statistics and correlations of mothers’ and fathers’ coparenting alliance and responsive parenting indicators and latent variables.

Variable		1	2	3	4	5	6	7	8	9	10	11	12	13	14	15	16	17	18	19	20
**Mothers’ reports of coparenting alliance at 15 months**																					
1	Good parent	–																			
2	Communication	0.48***	–																		
3	Good judgment	0.41***	0.50***	–																	
4	Job easier	0.36***	0.45***	0.49***	–																
5	Good team	0.43***	0.60***	0.53***	0.56***	–															
6	Handle children	0.42***	0.44***	0.50***	0.47***	0.51***	–														
7	Solve problems	0.37***	0.49***	0.56***	0.53***	0.62***	0.48***	–													
8	Personal sacrifice	0.40***	0.43***	0.38***	0.45***	0.53***	0.42***	0.49***	–												
9	Like talking	0.47***	0.58***	0.50***	0.47***	0.64***	0.47***	0.55***	0.48***	–											
10	Pays attention	0.46***	0.41***	0.47***	0.53***	0.51***	0.54***	0.50***	0.49***	0.48***	–										
**Fathers’ reports of coparenting alliance at 15 months**																					
11	Good parent	0.15**	0.07	−0.01	0.07	0.11*	0.12*	0.12**	0.17***	0.06	0.11*	–									
12	Communication	0.06	0.06	−0.02	0.09	0.10*	0.10*	0.08	0.10*	0.01	0.07	0.37***	–								
13	Good judgment	0.10*	0.00	0.05	0.11*	0.14**	0.14**	0.18**	0.13**	0.05	0.09*	0.54***	0.50***	–							
14	Job easier	0.10*	0.04	0.03	0.04	0.06	0.06	0.05	0.06	0.07	0.08	0.36***	0.39***	0.39***	–						
15	Good team	0.13**	0.08	0.12*	0.18***	0.24***	0.24***	0.22***	0.18**	0.14**	0.18**	0.46***	0.51***	0.55***	0.48***	–					
16	Handle children	0.09	0.10	0.01	0.11*	0.11*	0.11*	0.13**	0.11	0.06	0.12*	0.50***	0.43***	0.62***	0.48***	0.57***	–				
17	Solve problems	0.01	0.03	−0.02	0.10*	0.03	0.03	0.09	0.14**	0.03	0.04	0.37***	0.48***	0.45***	0.39***	0.49***	0.51***	–			
18	Personal sacrifice	0.14**	0.04	0.04	0.12*	0.16***	0.16***	0.17***	0.17***	0.08	0.15**	0.56***	0.42***	0.55***	0.52***	0.61***	0.63***	0.56***	–		
19	Like talking	0.11*	0.05	0.08	0.15*	0.14**	0.14**	0.19***	0.21***	0.10	0.12**	0.40***	0.45***	0.58***	0.44***	0.56***	0.57***	0.50***	0.56***	–	
20	Pays attention	0.16***	0.07	0.10*	0.05	0.11*	0.11*	0.11*	0.13**	0.10*	0.07	0.44***	0.32***	0.45***	0.50***	0.49***	0.59***	0.45***	0.64***	0.54***	–
*M*		4.76	4.61	4.60	4.42	4.60	4.53	4.60	4.66	4.67	4.72	4.82	4.67	4.74	4.65	4.71	4.74	4.66	4.77	4.67	4.77
*SD*		0.44	0.56	0.60	0.75	0.62	0.64	0.61	0.61	0.55	0.51	0.39	0.53	0.49	0.59	0.49	0.45	0.53	0.45	0.53	0.48
**Mothers’ reports of responsive parenting at 36 months**																					
21	Sensitivity	0.15**	0.01	0.07	0.03	0.06	0.07	0.09	0.08	0.07	0.07	0.05	0.05	0.12**	0.09	0.13**	0.08	0.00	0.07	0.13**	0.11*
22	Positive regard	0.15**	0.04	0.16***	0.12**	0.16***	0.12**	0.12*	0.16***	0.13**	0.10*	0.13**	0.06	0.16***	0.10*	0.17***	0.10*	0.03	0.14**	0.12**	0.11*
23	Cognitive stimulation	0.14**	0.03	0.06	0.07	0.07	0.08	0.08	0.08	0.08	0.07	0.04	−0.01	0.07	0.02	0.02	0.01	0.02	0.05	0.01	0.02
24	Intrusiveness	−0.06	0.04	0.05	0.03	0.00	0.02	0.00	−0.02	0.00	0.00	−0.10*	−0.10*	−0.07	−0.07	−0.09	−0.07	0.00	−0.08	−0.10*	−0.11*
25	Negative regard	0.02	0.04	−0.01	−0.04	−0.03	0.00	−0.01	−0.03	−0.02	−0.03	0.01	−0.03	−0.09	−0.09	−0.07	0.00	0.04	−0.07	−0.10*	−0.08
26	Detachment	−0.08	−0.07	−0.10*	−0.06	−0.09	−0.05	−0.10*	−0.05	−0.08	−0.05	0.00	0.05	−0.08	−0.07	−0.08	−0.10*	0.03	−0.03	−0.08	−0.06
**Fathers’ reports of responsive parenting at 36 months**																					
27	Sensitivity	0.14**	0.09*	0.06	0.07	0.08	0.09*	0.09	0.07	0.07	0.05	−0.01	−0.03	0.02	0.05	0.12*	0.05	0.01	0.04	0.11	0.06
28	Positive regard	0.13**	0.05	0.03	0.03	0.06	0.11*	0.02	0.02	0.05	0.07	0.05	−0.02	−0.01	0.00	0.05	0.01	−0.11*	0.04	0.07	0.07
29	Cognitive stimulation	0.09	−0.05	0.09	0.08	0.08	0.13**	0.08	0.06	0.04	0.10*	0.09	0.00	0.14**	−0.04	0.08	0.12**	−0.02	0.11*	0.10*	0.08
30	Intrusiveness	−0.17	−0.07	0.02	−0.02	−0.05	−0.07	−0.06	−0.01	−0.06	−0.04	0.01	0.07	−0.01	−0.05	−0.04	0.03	0.08	0.05	−0.01	0.01
31	Negative regard	−0.03	−0.07	0.00	−0.03	−0.03	−0.10*	−0.07	−0.02	−0.06	−0.02	0.06	0.09	−0.01	0.02	−0.06	−0.02	0.06	0.02	−0.10*	−0.05
32	Detachment	−0.07	−0.10*	−0.07	−0.04	−0.07	−0.06	−0.05	−0.02	0.04	−0.02	0.03	0.01	0.02	−0.07	−0.08	−0.03	−0.03	−0.05	−0.11*	−0.08
33	Material hardship	−0.02	−0.09	0.03	−0.04	−0.02	0.00	−0.05	−0.03	−0.04	−0.07	−0.03	0.06	0.04	−0.01	−0.01	−0.02	−0.04	0.00	0.03	−0.03
34	Child prosocial behaviors	0.13**	0.04	0.06	−0.02	0.04	0.06	0.02	0.06	0.04	0.05	0.17***	0.00	0.15**	−0.01	0.06	0.09	0.01	0.11*	0.06	0.12*
*M*		4.76	4.61	4.60	4.42	4.60	4.53	4.60	4.66	4.67	4.72	4.82	4.67	4.74	4.65	4.71	4.74	4.66	4.77	4.67	4.77
*SD*		0.44	0.56	0.60	0.75	0.62	0.64	0.61	0.61	0.55	0.51	0.39	0.53	0.49	0.59	0.49	0.45	0.53	0.45	0.53	0.48

Variable		21	22	23	24	25	26	27	28	29	30	31	32	33	34
**Mothers’ reports of responsive parenting at 36 months**															
21	Sensitivity	–													
22	Positive regard	0.64***	–												
23	Cognitive stimulation	0.39***	0.50***	–											
24	Intrusiveness	−0.68***	−0.33***	−0.10*	–										
25	Negative regard	−0.49***	−0.032***	−0.11*	0.52***	–									
26	Detachment	−0.60***	−0.43***	−0.30***	0.22***	0.31***	–								
**Fathers’ reports of responsive parenting at 36 months**															
27	Sensitivity	0.24***	0.19**	0.16***	−0.15**	−0.07	−0.18**	–							
28	Positive regard	0.16***	0.14*	0.12*	−0.14**	−0.13**	−0.07	0.63***	–						
29	Cognitive stimulation	0.19***	0.24***	0.27***	−0.16***	−0.07	−0.10*	0.41***	0.43***	–					
30	Intrusiveness	−0.15**	−0.12**	−0.14**	0.13**	0.06	0.07	−0.63***	−0.33***	−0.14**	–				
31	Negative regard	−0.21***	−0.12**	−0.13**	0.13**	0.21***	0.15**	−0.51***	−0.35***	−0.11*	0.51***	–			
32	Detachment	−0.18***	−0.14**	−0.10*	0.12**	0.12**	0.18**	−0.59***	−0.42***	−0.30***	0.17***	0.30***	–		
33	Material hardship	0.05	0.02	−0.01	−0.03	0.01	−0.01	0.04	0.06	0.05	−0.03	−0.02	−0.04	–	
34	Child prosocial behaviors	0.21***	0.24***	0.28***	−0.14**	−0.01	−0.13**	0.19***	0.12*	0.22***	−0.11*	−0.02	−0.05	0.05	–
*M*		4.69	4.40	4.15	2.97	2.13	2.46	4.62	4.35	4.10	3.02	2.05	2.39	1.35	2.37
*SD*		1.10	0.98	1.11	1.13	0.96	1.06	1.07	0.95	1.11	1.13	1.02	1.05	0.50	0.51

*p < 0.05; **p < 0.01; ***p < 0.001.

#### Preliminary Analyses and Data Reduction

Preliminary analyses involved exploratory factor analysis (EFA) to examine the number of factors underlying indices of mothers’ and fathers’ observed parenting behaviors. Eigenvalues were used to determine the number of factors. According to Kaiser’s criterion, factors with eigenvalues equal or higher than 1 can be retained (Kaiser, 1960). Separate unrotated principal factor EFAs were conducted for mothers and fathers, using each parent’s six parenting behaviors (i.e., sensitivity, positive regard, negative regard, cognitive stimulation, intrusiveness, and detachment) as individual items. For both parents, EFA results suggested a single factor model with the eigenvalues of the first factors being 2.59 for mothers 2.52 for fathers. All subsequent factors had eigenvalues less than 1. These first factors for mothers and fathers accounted for 90.19% and 93.19% of the total variance of the parenting items, respectively.

#### Building Latent Variables

Given the nature of the longitudinal and multiple reporter data available, analyses were designed in steps for purposes of model building. Building the model of interest from the smallest specified pieces ensures that all the pieces in the model are appropriately specified and fit the data well (Kline, 2016). Informed by the results of the EFA, a single factor CFA model was first tested with all six parenting variables for both parents. Models for both mothers and fathers converged normally, with fit indices indicating decent model fit and all factor loadings above the absolute value of 0.42 (for details, see Tables 3, 4). Next, a separate CFA was conducted to build a latent variable representing couple-level coparenting relationship quality variable (see also Lee et al., 2020). Because each parent reported on the other parent’s coparenting (e.g., “I believe my child’s other parent is a good parent”) rather than their own coparenting, both mothers’ and fathers’ reports of the coparenting relationship were used to create a second-order, couple-level latent variable to assess the dyadic nature of the coparenting construct. This process involved creating first-order coparenting latent variables for mothers and fathers using individual coparenting items reported by mothers and fathers. That is, two first-order coparenting latent variables were built, one for mothers and another for fathers. Models for both parents converged normally and had good fit to the data (Table 3). Factor loadings for individual coparenting items were all above 0.58 for both parents (Table 4). The two first-order coparenting latent variables were then used to create a single second-order coparenting latent variable that represented coparenting alliance present at the couple level instead of the individual parent level. Following recommendations for conducting dyadic analysis within a structural equation modeling (SEM) framework (Gonzalez and Griffin, 2012), we fixed the loadings for mothers’ and fathers’ first-order coparenting latent variables to be equal at 1. The residual variances of these first-order latent variables were also fixed to be equal. These constraints were imposed to reflect mothers’ and fathers’ equal contributions to the dyadic coparenting latent variable. Once more, the model with the second-order coparenting latent variable converged normally and had good fit to the data (see Tables 3, 4). Finally, a model combining the second-order coparenting latent variable with mothers’ and fathers’ responsive parenting latent variables was built and tested. This final combined model converged normally and had good fit to the data as shown in Table 3.

**TABLE 3:** Fit indices of individual confirmatory factor analysis models.

Model	*df*	χ^2^	*p*	RMSEA	90% CI	CFI	SRMR
First-order coparenting by mothers	35	54.26	<0.001	0.05	(0.02, 0.07)	0.98	0.03
First-order coparenting by fathers	35	66.35	<0.001	0.06	(0.04, 0.09)	0.97	0.04
Second-order coparenting by couples	190	2596.97	<0.001	0.04	(0.03, 0.05)	0.97	0.04
Mothers’ responsive parenting	15	1105.34	<0.001	0.07	(0.03, 0.11)	0.99	0.03
Fathers’ responsive parenting	15	1019.68	<0.001	0.07	(0.03, 0.11)	0.99	0.03
Mothers’ responsive parenting and fathers’ responsive parenting combined	66	2243.73	<0.001	0.05	(0.04, 0.07)	0.98	0.05
Second-order coparenting and parents’ responsive parenting combined	451	660.79	<0.001	0.04	(0.03, 0.04)	0.96	0.05

*RMSEA, Root Mean Square Error Approximation; CI, Confidence Interval; CFI, Comparative Fit Index; SRMR, Standardized Root Mean Square Residuals.*

**TABLE 4:** Measurement model: Factor loadings for latent variables.

Indicator	Unstandardized estimate	*SE*	*p*	Standardized estimate
**Coparenting alliance at 15 months**				

**First-order coparenting by fathers**				
CO1A: Child’s other parent is a good parent	1.00	–	–	0.64
CO1B: Other parent and I communicate well	1.24	0.12	<0.001	0.59
CO1C: Feel good about other parent judgment	1.43	0.11	<0.001	0.74
CO1D: Other parent makes parenting job easier	1.45	0.12	<0.001	0.62
CO1E: Other parent and I are a good team	1.49	0.13	<0.001	0.76
CO1F: Other parent knows how to handle child	1.42	0.11	<0.001	0.78
CO1G: We work a good solution together	1.35	0.12	<0.001	0.66
CO1H: Other parent willing to sacrifice	1.47	0.12	<0.001	0.82
CO1I: Look forward to talking with other parent	1.51	0.14	<0.001	0.74
CO1J: Other child pays attention to child	1.34	0.13	<0.001	0.71
**First-order coparenting by mothers**				
CO1A: Child’s other parent is a good parent	1.00	–	–	0.58
CO1B: Other parent and I communicate well	1.53	0.17	<0.001	0.69
CO1C: Feel good about other parent judgment	1.64	0.21	<0.001	0.69
CO1D: Other parent makes parenting job easier	1.93	0.22	<0.001	0.67
CO1E: Other parent and I are a good team	1.95	0.21	<0.001	0.80
CO1F: Other parent knows how to handle child	1.71	0.16	<0.001	0.68
CO1G: We work a good solution together	1.80	0.19	<0.001	0.74
CO1H: Other parent willing to sacrifice	1.51	0.17	<0.001	0.65
CO1I: Look forward to talking with other parent	1.66	0.15	<0.001	0.75
CO1J: Other child pays attention to child	1.35	0.15	<0.001	0.69
**Second-order coparenting by couples**				
First-order coparenting by mothers	1.00	–	–	0.45
First-order coparenting by fathers	1.00	–	–	0.45

**Responsive parenting at 36 months**				

**Fathers’ responsive parenting**				
Sensitivity	1.00	–	–	0.98
Detachment	−0.60	0.05	<0.001	−0.60
Positive regard	0.59	0.05	<0.001	0.64
Negative regard	−0.53	0.06	<0.001	−0.54
Cognitive stimulation	0.45	0.06	<0.001	0.43
Intrusiveness	−0.56	0.06	<0.001	−0.51
**Mothers’ responsive parenting**				
Sensitivity	1.00	–	–	0.95
Detachment	−0.64	0.07	<0.001	−0.63
Positive regard	0.63	0.05	<0.001	0.67
Negative regard	−0.46	0.06	<0.001	−0.50
Cognitive stimulation	0.43	0.05	<0.001	0.42
Intrusiveness	−0.56	0.06	<0.001	−0.52

**Correlated errors**				

Fathers’ detachment and intrusiveness	−0.17	0.04	<0.001	−0.21
Fathers’ negative regard and intrusiveness	0.28	0.04	<0.001	0.33
Fathers’ positive regard and cognitive stimulation	0.16	0.04	<0.001	0.21
Fathers’ sensitivity and intrusiveness	−0.16	0.06	0.013	−0.71
Mothers’ positive regard and cognitive stimulation	0.21	0.04	<0.001	0.30
Mothers’ negative regard and intrusiveness	0.25	0.04	<0.001	0.32
Mothers’ detachment and intrusiveness	−0.11	0.04	0.009	−0.14
Mothers’ sensitivity and intrusiveness	−0.24	0.06	<0.001	−0.73
Fathers’ negative regard and mothers’ negative regard	0.15	0.04	<0.001	0.20
Fathers’ cognitive stimulation and mothers’ cognitive stimulation	0.15	0.04	0.001	0.15
Fathers’ responsive parenting and mothers’ responsive parenting	0.22	0.06	<0.001	0.21

#### Building the Structural Equation Model

The study used SEM as its main analytic method to test paths specified in the conceptual model (Figure 1). Specifically, the associations between family material hardship and children’s prosocial behaviors mediated by coparenting alliance and mothers’ and fathers’ responsive parenting were tested. The SEM models included the responsive parenting latent variables for mothers and fathers, and the couple-level coparenting alliance latent variable built previously. Material hardship and children’s prosocial behaviors were composites that served as observed variables in the model. SEM analyses were conducted using the R package *lavaan* (Rosseel, 2012) to estimate the models. Due to non-normality in some of the variables (mainly the coparenting alliance items), the robust maximum likelihood (MLR) estimator was used, which produces a scaled Yuan-Bentler chi-square statistic test and Huber-White standard errors that are robust to non-normality in the data (Huber, 1967; Yuan and Bentler, 2000). Indirect effects were tested by estimating Monte Carlo confidence intervals, which involves repeating thousands of random draws from the joint distribution of parameter estimates of interest (*a* and *b*) to produce a sampling distribution of an indirect effect (*ab*). This information is then used to estimate confidence intervals for the indirect effect (Preacher and Selig, 2012). Monte Carlo confidence intervals yield comparable results as the non-parametric bootstrapped confidence intervals in simulation studies, with similar advantages (i.e., no distributional assumptions about the indirect effect and thus allowing for asymmetry in its confidence interval) (Preacher and Selig, 2012). The null hypothesis that no indirect effect exists is tested by examining whether the Monte Carlo confidence interval includes a zero. If the confidence interval does not include a zero, then we can claim that an indirect effect is different from zero (Dearing and Hamilton, 2006; Preacher and Hayes, 2008).

Model fit was evaluated using several fit indices (see Kline, 2016), including Root Mean Square Error Approximation (RMSEA; Steiger, 1990; <0.06 for good fit); 90% confidence intervals (CIs) of RMSEA (<0.05 for lower bound for good fit; Kenny, 2015); Comparative Fit Index (CFI; Bentler, 1990; >0.95 for good fit); and Standardized Root Mean Square Residuals (SRMR; Hu and Bentler, 1999; <0.05 for good fit). The chi-square test of significance was reported but not primarily relied upon to assess model fit because it has been shown to be highly sensitive to sample size (Kline, 2016). Children’s gender was examined as a moderator, given prior literature indicating possible gender differences in children’s prosocial behaviors (Rose and Rudolph, 2006). Measurement invariance tests and multigroup analyses were conducted to examine differences in family processes when the focal child was either a boy or girl.

Because the sample was drawn from a larger intervention study and because BSF random assignment status was significantly correlated with one of the study variables (i.e., coparenting alliance as reported by fathers), preliminary analyses examined BSF random assignment status as a moderator of the main SEM models. Upon establishing configural and metric invariances, comparison between the constrained model that fixed all regression paths to be equal across BSF intervention and control group families and an unconstrained model that allowed all regression paths to vary across the two groups showed that the two models were not significantly different from each other, Δχ^2^ (30) = 37.73, *p* = 0.157. These results suggest that models did not differ across BSF families in the intervention and control groups and that the unconstrained model should be retained. Therefore, we report the analyses for the larger combined sample of BSF families.

#### Missing Data

Stata Version 15.1 (StataCorp, 2017) was used to engage in missing data analysis. Missingness pattern analysis results showed that missing data were <1% for all main and sociodemographic control variables. The only exception was couples’ relationship length variable which were missing 2.43% of the cases. To account for missing data, full information maximum likelihood (FIML) was used in the SEM models. FIML estimates parameters by maximizing the sample and using all available data (Kline, 2016) and has been shown to produce less biased and more efficient estimates than other missing data methods (e.g., listwise deletion) (Allison, 2003).

## Results

Sample characteristics are presented in Table 1. Descriptive statistics and correlations between main study variables are presented in Table 2.

### Structural Equation Modeling Results

**FIGURE 2:**
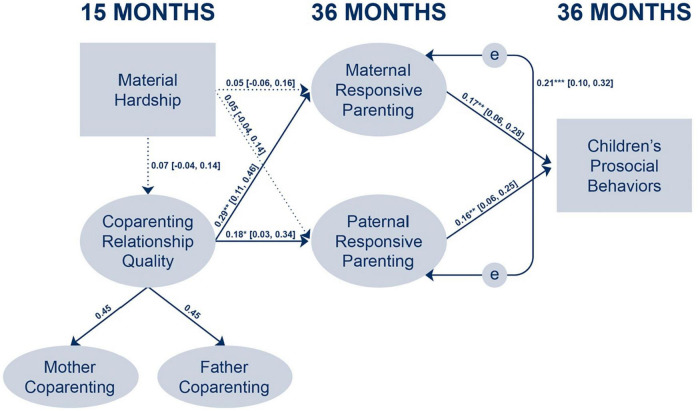
Results of the final structural equation model. *X*^2^ (928) = 1374.60, *p* < 0.001, RMSEA = 0.03, 90% CI (0.03, 0.04), CFI = 0.93, SRMR = 0.05. The model included the following sociodemographic control variables: White, Latinx, other, only one parent has a high school diploma, both parents have a high school diploma, fathers’ employment status, mothers’ depressive symptoms, fathers’ depressive symptoms, fathers’ multiple partner fertility, fathers’ involvement in caregiving, BSF random assignment status, and BSF program site location. Maternal depressive symptoms (β = 0.13, *p* = 0.006) and paternal depressive symptoms (β = 0.17, *p* = 0.003) were significantly associated with higher levels of families’ material hardship. Being Latinx (β = –0.35, *p* = 0.001), maternal depressive symptoms (β = –0.34, *p* < 0.001), and paternal depressive symptoms (β = –0.30, *p* < 0.001) were significantly associated with lower levels of coparenting alliance. Being randomly assigned to the BSF intervention group (β = 0.24, *p* = 0.001) was associated with higher levels of coparenting alliance. Standardized regression coefficients and 95% confidence intervals (in brackets) are shown. Dotted lines indicate non-significant paths. *p < 0.05, **p < 0.01, ***p < 0.001.

The main SEM model examined links between families’ material hardship, couple-level coparenting alliance, mothers’ and fathers’ responsive parenting and children’s prosocial behaviors. As shown in Figure 2, structural paths were estimated between (a) material hardship and coparenting alliance; (b) material hardship and mothers’ responsive parenting; (c) material hardship and fathers’ responsive parenting; (d) coparenting alliance and mothers’ responsive parenting; (e) coparenting alliance and fathers’ responsive parenting; (f) mothers’ responsive parenting and children’s prosocial behaviors; and (g) fathers’ responsive parenting and children’s prosocial behaviors. The SEM model converged normally, and the model had good fit to the data, χ^2^ (928) = 1374.60, *p* < 0.001, RMSEA = 0.03, 90% CI (0.03, 0.04), CFI = 0.93, SRMR = 0.05.

Figure 2 also shows that material hardship at 15 months was not significantly linked with any of the main variables, including the coparenting alliance at 15 months [β = 0.07, *p* = 0.353, 95% CI (−0.04, 0.14)], maternal responsive parenting at 36 months [β = 0.05, *p* = 0.411, 95% CI (−0.06, 0.16)], and paternal responsive parenting at 36 months [β = 0.05, *p* = 0.282, 95% CI (−0.04, 0.14)]. Coparenting alliance at 15 months was a significant positive predictor of both maternal and paternal responsive parenting at 36 months: Maternal responsive parenting, β = 0.29, *p* = 0.001, 95% CI (0.11, 0.46), and paternal responsive parenting, β = 0.18, *p* = 0.020, 95% CI (0.03, 0.34). Maternal responsive parenting at 36 months subsequently was a significant positive predictor of children’s prosocial behaviors at 36 months, β = 0.17, *p* = 0.002, 95% CI (0.06, 0.28). Similarly, paternal responsive parenting at 36 months was a significant positive predictor of children’s prosocial behaviors at 36 months β = 0.16, *p* = 0.001, 95% CI (0.06, 0.25).

Tests of indirect effects were conducted by estimating the Monte Carlo confidence intervals for the indirect effects. We used a random draw of 100,000 samples to obtain the sampling distributions of the six main indirect effects. This included the indirect effects of (1) maternal responsive parenting as a mediator between material hardship and children’s prosocial behaviors; (2) maternal responsive parenting as a mediator between coparenting alliance and children’s prosocial behaviors; (3) paternal responsive parenting as a mediator between material hardship and children’s prosocial behaviors; (4) paternal responsive parenting as a mediator between coparenting alliance and children’s prosocial behaviors; (5) coparenting alliance as a mediator between material hardship and maternal responsive parenting; and (6) coparenting alliance as a mediator between material hardship and paternal responsive parenting. Examination of the Monte Carlo confidence intervals showed that only those for the second and fourth indirect effects involving coparenting alliance, responsive parenting, and children’s prosocial behaviors did not include a zero and indicated significant indirect effects: Maternal responsive parenting as a mediator between coparenting alliance and children’s prosocial behaviors, indirect effect = 0.22, 95% CI (0.04, 0.48); and paternal responsive parenting as a mediator between coparenting alliance and children’s prosocial behaviors, indirect effect = 0.13, 95% CI (0.01, 0.29). The Monte Carlo confidence intervals for all other indirect effects did include a zero and therefore were not significant: Maternal responsive parenting as a mediator between material hardship and children’s prosocial behaviors, indirect effect = 0.01, 95% CI (−0.01, 0.03); paternal responsive parenting as a mediator between material hardship and children’s prosocial behaviors, indirect effect = 0.01, 95% CI (−0.01, 0.03); coparenting alliance as a mediator between material hardship and maternal responsive parenting, indirect effect = 0.04, 95% CI (−0.05, 0.14); and coparenting alliance as a mediator between material hardship and paternal responsive parenting, indirect effect = 0.03, 95% CI (−0.03, 0.10). Together, these results confirmed that maternal and paternal responsive parenting mediated the associations between the coparenting alliance and higher levels of children’s prosocial behaviors.

### Moderation Analyses

Girls in our sample did not exhibit significantly higher prosocial behaviors than boys (girls: *M* = 2.39, *SD* = 0.49; boys: *M* = 2.35, *SD* = 0.53) based on one-way analysis of variance results, *F*(1) = 0.69, *p* = 0.407. With that in mind, we still proceeded to examined children’s gender as a potential moderator. Measurement invariance was first conducted using children’s gender as a grouping variable. Both configural and metric invariance were tested. Only configural invariance was present in the latent variables across boys and girls, and the chi-square test result comparing the constrained model that fixed all regression paths to be equal across boys and girls to an unconstrained model that allowed all regression paths to vary across boys and girls showed that the two models were not significantly different from each other, Δχ^2^ (32) = 32.45, *p* = 0.445. Thus, our results suggested that processes linking material hardship, coparenting alliance, and mothers’ and fathers’ responsive parenting, and children’s prosocial behavior may not vary across families with boys and girls and that the unconstrained model should be retained.

## Discussion

The current study utilized a risk and resilience approach to understanding the effects of material hardship on preschoolers’ prosocial behaviors as mediated by supportive coparenting alliance and mothers’ and fathers’ responsive parenting. Using a sample of families from socioeconomically disadvantaged backgrounds, we tested three specific hypotheses based on the FSM and prior research. First, we hypothesized that material hardship would be associated with less supportive coparenting alliance and less responsive parenting for both mothers and fathers (H1). Next, a stronger coparenting alliance would predict more responsive maternal and paternal parenting (H2). Finally, maternal and paternal responsive parenting would be linked with higher levels of children’s prosocial behaviors and act as mediating pathways between coparenting and children’s prosocial behaviors (H3).

### Resilience Against the Adverse Effects of Material Hardship on Responsive Parenting

Although our results did not support the first hypothesis of the negative effects of material hardship on less supportive coparenting alliance and parental responsiveness, there was more support for the second and third hypotheses. The fact that material hardship did not appear to have an effect on both coparenting and parenting was surprising, as we expected that material hardship should affect family relations adversely as proposed by the FSM (Conger et al., 1992). Again, FSM posits that economic pressures stemming from negative economic events such as low family income lead to poorer interparental relationship quality, which is subsequently linked with less involved and nurturant parenting behaviors (Conger et al., 1992). However, from a resilience and risk perspective—which allows for understanding how certain positive family functioning may be protective against the negative impact of economic difficulties on families and children—it is possible that BSF families in our sample found ways to be resilient against the adverse effects of material hardship. Specifically, a strong positive coparenting relationship between BSF mothers and fathers may have served a source of resilience, buffering against the potentially negative effects material hardship could have had on subsequent parenting behaviors.

Relatedly, while the FSM proposes negative effects of material hardship on family functioning, research evidence with BSF families or families from similarly disadvantaged backgrounds show rather mixed findings in this area. The current study’s findings would appear both consistent and inconsistent with the results of such prior work examining the links between material hardship, coparenting alliance, and responsive parenting (Waller, 2012; Baker et al., 2018; Shelleby, 2018; LeBaron et al., 2020; Curran et al., 2021). For examples, our results are consistent with those of Curran et al. (2021) who used a cross-lagged panel analyses with 4,424 BSF families and found that material hardship at 15 months did not predict either parent’s coparenting alliance 36 months, suggesting that a strong sense of coparenting alliance may be robust against material hardship’s negative effects. However, our results are inconsistent with those of LeBaron et al. (2020), who found that for a BSF sample, material hardship at 15 months negatively predicted fathers’ (but not mothers’) reports of coparenting alliance at 36 months.

There are few reasons why our findings may be different from what others have found (i.e., LeBaron et al., 2020). For one, there are differences in sample characteristics across studies, even in cases where BSF families were the focus. For example, our sample included only mothers and fathers with complete observational data at 36 months, which meant most of these couples included a residential father living with both the mother and child given that home observations were not conducted with the majority of couples who were not residing together. LeBaron et al. (2020), on the other hand, included both residential and non residential father families, with nearly half of the families having non residential fathers. Further, mothers and fathers in the current analyses were living consistently together across the two times of measurement, which might suggest that these couples had a stronger coparenting alliance than those in LeBaron et al. (2020), and this more supportive coparental alliance may have protected couples against the negative effects of material hardship for those in the current study.

Another reason for the differences may pertain to statistical methods and analyses. Given the nature of the coparenting alliance that involves both parents, we employed a latent variable approach to create a measure of dyadic coparenting, taking both mothers’ and fathers’ reports into consideration. LeBaron et al. (2020) chose to use separate reports of mothers’ and fathers’ coparenting in their analysis. The effects of material hardship may differ for men and women in the family based on the differing societal expectations of gendered roles for mothers and fathers, with mothers often assuming more child care responsibilities and fathers more responsible for the family’s economic security. As such, parents may be more or less vulnerable to the effects of material hardship when considering mothers and fathers separately that we do not see when considering coparenting as a dyadic construct. Whatever the exact reason for differences in results between studies, our results suggest that BSF couples focused on working together as a coparenting team may be resilient against stressors and risk stemming from poverty.

### Associations Between the Coparenting Alliance, Mothers’ and Fathers’ Responsive Parenting and Children’s Prosocial Behaviors

We found support for our second hypothesis that a supportive coparenting alliance at 15 months predicted more responsive parenting for both mothers and fathers at 36 months (H2), as well as our third hypothesis that mothers’ and fathers’ responsive parenting predicted higher levels of children’s prosocial behaviors at 36 months (H3). In line with a risk and resilience approach to testing the FSM, the coparenting alliance—in which two parents coordinate and cooperate in their parenting roles—seemed to have acted as the “executive subsystem” that improves family functioning and thus children’s developmental outcomes (Minuchin, 1988; Cox et al., 2001), including those amongst socioeconomically disadvantaged families (Jones et al., 2005; Shook et al., 2010; Barnett et al., 2011; Fagan and Palkovitz, 2011; Lee et al., 2020).

For example, for mothers from low income backgrounds, positive coparenting in the form of support and communication has been linked with increased levels of mothers’ positive perceptions of fathers’ engagement (e.g., childcare and play activities with the children) (Fagan and Palkovitz, 2011) and mothers’ supportive parenting behaviors toward the child characterized by high levels of sensitivity, cognitive stimulation, and positive regard (Barnett et al., 2011, 2012; Cabrera et al., 2012). Similarly, when parents cooperate as a coparenting team, fathers with low income were more likely to spend time with their children (Coley and Chase-Lansdale, 1999), engage in caregiving activities (Lee et al., 2020), provide instrumental support, and communicate with the mother about their children (Hohmann-Marriott, 2011). In light of such prior research, again our findings suggest that a strong coparenting alliance may be beneficial to both parents and children in that it serves as a source of resilience for families facing material hardship. Should parents with low income work to maintain supportive coparenting relationships, even in economically challenging circumstances, mothers and fathers can still engage in responsive and stimulating parenting practices that ultimately benefit their children’s socioemotional development.

Moreover, in the current study, the coparenting alliance between mothers and fathers had an indirect effect on children’s prosocial behavior through promoting both mothers’ and fathers’ responsive parenting practices. This is consistent with our third hypothesis (H3) and prior research showing similar mechanisms by which coparenting is positively linked to children’s developmental outcomes (Cabrera et al., 2012; Yan et al., 2018). For example, Cabrera et al. (2012) used a sample from ECLS-B to show that for both married and cohabiting families, coparenting communication between mothers and fathers when children were 24 months old was concurrently linked with higher levels of mothers’ supportive parenting, which was then linked with higher levels of children’s social skills (e.g., playing with other children, trying to understand others) when the children were 4 years old. The researchers did not test fathers’ supportive parenting, however. Notably, in our study, while maternal responsive parenting had an indirect effect larger in magnitude than paternal responsive parenting, the significant indirect effect of paternal responsive parenting suggests that fathers make an important contribution to their preschoolers’ prosocial development even after accounting for maternal effects. In other words, both mothers and fathers seemed to play a role in promoting their children’s development of prosocial behaviors. Given the limited research in this area, especially using data from both mothers and fathers from low income backgrounds, our finding makes an important contribution to better understanding processes underlying coparenting and young children’s socioemotional development in such families.

In summary, by taking a risk and resilience approach to testing the FSM, results from the current study suggest that coparenting alliance plays a protective role amidst risk ensued by material hardship. That is, even in economically challenging circumstances when mothers and fathers with low income work together toward having supportive coparenting relationships (i.e., a source of resilience for the family), they may be able to engage in responsive parenting practices. Importantly, the supportive coparenting relationship mothers and father shared in our sample seemed to have worked as an executive subsystem that contributed to both parents’ positive parenting behaviors that ultimately supported their young children’s socioemotional development. For these families, having a strong alliance between mothers and fathers around coparenting served as a source of resilience and thus played a protective role against the risks of experiencing material hardship.

### Limitations

There are several limitations to the current study that need to be noted. Although food insecurity is a key aspect of material hardship, we were unable to include it as part of the measure of material hardship because the BSF project did not collect information on the food needs BSF families faced. Further, results cannot be generalized to larger groups of families with low income because BSF families were a unique group willing to participate in a marriage and relationship improvement intervention. In addition, only a subset of families with complete observational parenting data for both mothers and fathers were used here, and observational data were mainly collected and available for couples living in the same household. These families were likely to have been highly motivated to strengthen their coparental and parent-child relationships from the beginning. Parents with low income are diverse, and therefore, family processes may playout differently depending on the residential status of the father, as well as families’ race and ethnicity (Lee et al., 2020). Future studies may want to consider using family structure, such as fathers’ residential status, and race and ethnicity as possible moderators when looking at the effects of material hardship on family relationship functioning and children’s outcomes. Despite these limitations, the current study contributes to the literature by taking a risk and resilience approach to family stress brought on by economic hardship to understand underlying family processes in a large and racially diverse sample of two-parent families with young children.

### Implications for Family Strengthening Policies and Practices

The findings have implications for family strengthening policies and practices as well. As it pertains to the national Healthy Marriage and Responsible Fatherhood (HMRF) policy initiatives and subsequent responsible fatherhood programs, one of the goals of these policy and programmatic efforts has been to help fathers overcome barriers (i.e., unemployment, child support orders, relationship instability, access to parenting education) so they may engage in nurturant parenting (Patnaik and Avellar, 2020). The main idea is that by improving fathers’ parenting, responsible fatherhood programs can ultimately benefit children. Results of the current study suggest that focusing on strengthening the coparenting alliance in the face of economic stressors may be fruitful, as a strong coparenting alliance seemed to emerge as a protective factor that promoted responsive fathering (and mothering). Responsible fatherhood programs may want to consider focusing on strengthening the sense of solidarity and teamwork around coparenting between mothers and fathers with low income.

Prior large demonstration projects-funded by the Administration of Children and Families at the U.S. Department of Health and Human Services, including the BSF project and the more recent Parents and Children Together (PACT)-have not given much attention to strengthening the coparenting alliance nor to supporting parents to work together as a parental team to raise their children to the same extent that these programs have focused on couples’ relationships and marriages (Wood et al., 2014; Zaveri et al., 2015; Avellar et al., 2018). For instance, BSF’s main goal was to improve marriage rates among couples with low income expecting a child and thus a focus on coparenting was almost non-existent in the curricula programs used as part of the project (Wood et al., 2014). PACT’s main goals were to improve adult and father-child relationships. While the programs included coparenting content in their curricula, much of it seemed to be delivered in a single workshop or formed only a small part of many lessons provided under large curricular themes, such as “Parenting and Fatherhood” or “Relationships and Marriage” (Zaveri et al., 2015). Much like BSF, the PACT project placed a larger focus on improving romantic relationships over coparenting relationships, with workshops focusing on conflict management, communication, and the impact of parents’ intimate relationships on children (Zaveri et al., 2015).

Not surprisingly, the PACT evaluation did not have any program effects on coparenting, including coparenting alliance, and recommendations for future projects included a focus on improving coparenting to promote father involvement (Avellar et al., 2018). Smaller scale studies that primarily focus on implementing coparenting interventions—with curricula focusing on creating coparenting solidarity, sharing parenting responsibilities, and improving communication around parenting—have demonstrated program effectiveness in reducing coparenting conflict and improving parenting, including father involvement in caregiving activities (Fagan, 2008; Pruett et al., 2019). For example, Fagan (2008) conducted a randomized study of the Minnesota Early Learning Design coparenting program with young Black and Latinx couples and found positive program effects on mothers’ and fathers’ coparenting behaviors and fathers’ engagement in infant care. These results suggest that federally funded demonstration projects and responsible fatherhood programs aiming to improve fathers’ parenting will do well to focus on implementing programs specifically designed to strengthen the coparenting alliance between mothers and fathers.

Related to this is the importance of including mothers in responsible fatherhood programs, as researchers have suggested that coparenting aspects of these programs would be more effective if mothers were also the recipients of coparenting education and training (Cowan and Cowan, 1995; Fagan, 2008). Recently, McKee et al. (2020) reported that the most significant predictor of parent participation in an intervention directed to low-income parents of infants was the participation of the other parent. More broadly, coparenting typically involves a minimum of two caregivers and cannot be carried out alone. Programs trying to enhance coparenting relationships may need to reflect this dyadic and family systems nature of coparenting. That is, a coparenting intervention may need buy-in from both fathers and mothers for it to be effective in improving the coparenting alliance and thus benefit subsequent family processes. Although three out of four of the PACT programs encouraged mothers to join relationship workshops, they were often not well attended (Dion et al., 2015).

Programs like the Young Parenthood Program (YPP; Florsheim et al., 2012) and Supporting Fatherhood Involvement (SFI; Pruett et al., 2019) are promising examples of coparenting interventions that include both parents. A randomized controlled trial of YPP with adolescent fathers and mothers during the prenatal period showed positive direct effects on fathers’ engagement in childrearing, fathers’ reports of coparenting relationship quality (i.e., coparenting support and depth in dyadic relationship), and mothers’ reports of coparenting competence (i.e., capacity to retain a positive perspective on the coparenting relationship and engage in positive coparenting behaviors) when children were 18 months old (Florsheim et al., 2012). For responsible fatherhood programs to be successful, program staff may need to convince mothers (and fathers) that they play important roles in creating supportive coparenting alliances that benefit their parenting and, ultimately, their children’s wellbeing.

## Data Availability Statement

Restricted data were analyzed in this study, and the data can be found here: https://www.icpsr.umich.edu/web/ICPSR/studies/29781/datadocumentation.

## Ethics Statement

The Health Sciences and Behavioral Sciences Institutional Review Board at the University of Michigan approved the current study as secondary analysis of the Building Strong Families (BSF) data (HUM00145063). The participants provided their written informed consent to participate in the original BSF study.

## Author Contributions

JYL and BLV conceptualized the main ideas of the manuscript. JYL conducted the main analyses, reviewed the results, interpreted the results, and wrote the manuscript. BLV reviewed the results, provided support for interpreting the results, and assisted with writing the manuscript. SJL reviewed the results and provided support for interpreting the results. All authors contributed to the article and approved the submitted version.

## Conflict of Interest

The authors declare that the research was conducted in the absence of any commercial or financial relationships that could be construed as a potential conflict of interest.

## Publisher’s Note

All claims expressed in this article are solely those of the authors and do not necessarily represent those of their affiliated organizations, or those of the publisher, the editors and the reviewers. Any product that may be evaluated in this article, or claim that may be made by its manufacturer, is not guaranteed or endorsed by the publisher.
